# COVID-19 and Health Outcomes in People with Multiple Sclerosis: A Population-Based Study in Italy

**DOI:** 10.3390/life13051089

**Published:** 2023-04-27

**Authors:** Roberto Gnavi, Ilenia Eboli, Paolo Emilio Alboini, Sandra D’Alfonso, Roberta Picariello, Giuseppe Costa, Maurizio Leone

**Affiliations:** 1 Epidemiology Unit, ASL TO3 Regione Piemonte, 10095 Grugliasco, Italy; 2 Neurology Unit, Fondazione IRCCS Casa Sollievo della Sofferenza, 71013 San Giovanni Rotondo, Italy; 3 Department of Health Sciences, and CAAD, University of Eastern Piedmont, 28100 Novara, Italy; 4 Department of Biological and Clinical Sciences, University of Turin, 10128 Torino, Italy

**Keywords:** multiple sclerosis, COVID-19, outcome assessment, health care

## Abstract

People with multiple sclerosis (PWMS) are at high risk of being affected by the disruption of health services that occurred during the COVID-19 pandemic months. The aim of this study was to evaluate the effect of the pandemic on the health outcomes of PWMS. PWMS and MS-free residing in Piedmont (north-west of Italy) were identified from electronic health records and linked with the regional COVID-19 database, the hospital-discharge database, and the population registry. Both cohorts (9333 PWMS and 4,145,856 MS-free persons) were followed-up for access to swab testing, hospitalisation, access to the Intensive Care Unit (ICU), and death from 22 February 2020 to 30 April 2021. The relationship between the outcomes and MS was evaluated using a logistic model, which was adjusted for potential confounders. The rate of swab testing was higher in PWMS, but the positivity to infection was similar to that of MS-free subjects. PWMS had a higher risk of hospitalisation (OR = 1.74; 95% IC, 1.41–2.14), admission to ICU (OR = 1.79; 95% IC, 1.17–2.72), and a slight, albeit not statistically significant, increase in mortality (OR = 1.28; 95% IC, 0.79–2.06). Compared to the general population PWMS with COVID-19 had an increased risk of hospitalization and admission to the ICU; the mortality rate did not differ.

## 1. Introduction

Italy was the first European country to be affected by the Coronavirus Disease 2019 (COVID-19) pandemic. Since the first Italian death was recorded in February 2020, the organisation of health services has been severely affected, leading to a reduction in access to hospitals and outpatient care services [1]. Patients suffering from chronic diseases have faced increasing difficulties in coping with the disruption of their care pathways, which require periodical laboratory tests and the constant monitoring of therapies [2].

Multiple Sclerosis (MS) is a progressive, chronic disease requiring constant monitoring through specialist assessments and therapies. Thus, people with MS (PWMS) are at a high risk of being affected by restrictions caused by the pandemic [3,4].

Several case series have been published on the prevalence of COVID-19 infection in PWMS and their outcomes [5,6], speculating that PWMS are at higher risk for a severe course of COVID-19 [7,8,9] and raising concerns among patients. However, most of this information comes from cohorts of hospitalised patients [10], patients recruited in MS expert centres [7,11,12,13,14,15,16,17], or from electronic health records with selected population coverage [13], who are, thus, more prone to selection biases and the reduced generalisability of the results. To address this gap in knowledge, unselected population-based studies are needed.

In Italy, only one study has evaluated the use of healthcare services during the pandemic among PWMS, observing a disruption in rehabilitative therapy, home-based services, and adherence to Disease Modifying Therapies (DMT) [18], but no data were available on outcomes. Therefore, we aimed to evaluate, during the first 15 months of the pandemic, the effect of reduced care on several indicators, including both access to health services and outcomes, using linked health administrative data to compare the MS population with the MS-free population of an entire Northern Italian region.

## 2. Materials and Methods

### 2.1. Study Population and Data Sources

The study was conducted in Piedmont (a region in the northwest of Italy with approximately 4,300,000 inhabitants). All residents can rely on a universalistic public health system that includes all health care services, whose data are routinely collected in an automated system of databases. To comply with privacy laws, patients’ personal data are pseudo-anonymised, and all databases are enriched with a unique anonymous identifier, encrypted to protect the patient’s privacy, which allows a record linkage between them.

Using a previously validated algorithm that has shown to be highly reliable (sensitivity 95.9%, specificity 99.9%) [19], we identified PWMS residing in Piedmont on 22 February 2020: the date of the first death from COVID-19 recorded in Italy. Briefly, the algorithm was based on the deterministic record linkage of four data sources: hospital discharges with a primary or secondary diagnosis of MS; the drug prescriptions used for MS; subjects who obtained exemption from the co-payment of drugs and outpatient visits due to the diagnosis of MS; patient resident in long-term care facilities with a diagnosis of MS. Any subject recorded in at least one of the above databases was considered an MS case. Residents without MS on the same date and within the same range of age as that of PWMS were considered as an MS-free population.

From the beginning of the COVID-19 epidemic, a surveillance system was implemented in Piedmont, recording data from all residents undergoing both reverse transcriptase-polymerase chain reaction (PCR testing) and antigen tests (as soon as they came into use) for SARS-CoV-2. All personal data are pseudo-anonymised using the same procedures as described above. The MS and MS-free populations were, thus, linked to the COVID-19 database to identify those who resulted in being COVID-19 positive; finally, they were further linked to the regional hospital-discharge database (HD) and to the regional registry office. In this way, we were able to follow up with each patient in terms of hospitalisation and mortality.

### 2.2. Outcomes

We considered five separate outcomes that summarised the patients’ disease course during the epidemic from 22 February 2020 to 30 April 2021. We obtained information about testing for SARS-CoV-2 (outcome 1) and positive testing (outcome 2) from the surveillance system; a subject was considered “positive” in the case of either a positive PCR or antigen test (so-called rapid test) recorded on the surveillance system database. In the case of more than one positive test, we considered the first one; if a positive antigen test was followed by a negative PCR test (performed within 7 days from the rapid test), the patient was considered COVID19-free; in the case of a positive rapid test with no evidence of a subsequent PCR test, the patient was considered to be affected by COVID-19. The diagnosis of COVID-19 infection was confirmed by a PCR test for 95% of both MS and MS-free cases. Hospitalisation within 30 days after testing positive (outcome 3) was determined from record linkage with the HD. Among these patients, we identified those who were admitted to an intensive care unit (ICU) (outcome 4) based on whether the HD showed evidence of admission (or transfer) to an ICU or an ICD9 cm code referring to mechanical ventilation. Finally, we determined 30-day mortality after testing positive (outcome 5) by record linkage with the registry office.

### 2.3. Clinical Characteristics and Comorbidities

We categorised the patients according to their socio-demographics and two comorbidities (diabetes and Chronic Obstructive Pulmonary Disease—COPD), which proved strongly correlated to both higher rates of infection and worse outcomes, present on 22 February 2020. Age was categorised into 10-year age intervals: <30, 30–39, 40–49, 50–59, 60–69, and >69 years old. Individual educational level, which was available for 80% of subjects, was obtained by record linkage with the last national census or, for those who immigrated after the census date, with the educational level recorded in the HD database; this was classified into four classes: low (<8 years of education), medium (up to 12 years of education), high (>13 years of education) and missing. Information regarding diabetes or COPD was obtained using algorithms based on the record linkage of administrative data sources (HD, drug prescriptions, exemption form co-payment because of confirmed diagnosis) [20].

### 2.4. Statistical Analysis

For each of the five outcomes, we calculated the proportions (i.e., the crude prevalences) of the variables, and the differences in the baseline characteristics between PWMS and the MS-free population were evaluated using the χ^2^ test. To investigate the relationship between the outcomes and MS, we used a logistic model adjusted for potential confounders (sex, age, educational level, diabetes, and COPD); the results are presented as odds ratios (ORs) with 95% confidence intervals. All analyses were performed using SAS System version 9.4. SAS Institute Inc, Cary, NC, USA.

## 3. Results

At the beginning of the COVID-19 epidemic, 9333 PWMS and 4,145,856 subjects without MS, between 6 and 92 years old, resided in Piedmont.

Table 1 presents the characteristics of the two populations. Women made up two-thirds of MS patients, while, in the MS-free population, sex was evenly distributed. Similarly, age was differently distributed between PWMS, for which the most frequent classes were 40–49 and 50–59, and for the MS-free population, the most frequent classes were <30 years and ≥70 old. The educational level was higher among PWMS, but the MS-free population had a larger number of missing values. There were no differences in the prevalence of COPD between the two groups, while diabetes was more frequent in the MS-free population.

Table 2 shows crude prevalence rates for five outcomes in the two populations. PWMS were more likely to undergo swab testing (37.9% vs. 32.3%), especially women and younger subjects. COVID-19 positivity among tested individuals was similar between the two populations (24.5% vs. 24.3%), with slight differences in age and educational level but no differences due to comorbidity.

Considering the subjects who tested positive, the probability of hospitalisation was higher for PWMS of both sexes, all ages, and educational levels; there were no differences for patients affected by COPD, while those with diabetes were less likely to be hospitalised.

The two most severe outcomes, ICU admission and mortality, showed an opposite pattern: ICU admissions were slightly higher in PWMS (2.7% vs. 2.0%), but 30 days mortality (including in- and out-hospital) was lower (2.2% vs. 3.7%). Regarding the former, differences were evident only for sex (MS women more likely admitted) and diabetes (MS-free more likely admitted), while there were no differences for age, educational level, or COPD. As for mortality, statistically significant differences emerged for age (with different results depending on the age considered) and educational level.

**Figure 1:**
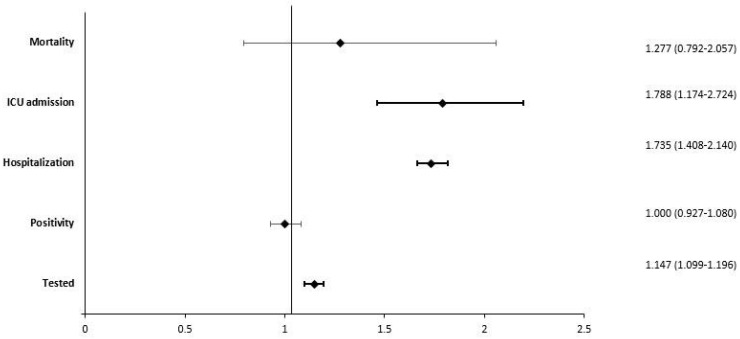
Forest plot showing adjusted odds ratio for five outcomes.

The multivariate model (Figure 1), which was adjusted for all the potential confounders we considered, confirmed that PWMS had a higher probability of being tested for COVID-19 (OR = 1.15; IC 95%: 1.10–1.20) but showed no differences in positivity when testing (OR = 1.00; IC 95%: 0.93–1.08). Among the positive individuals, PWMS had a higher risk of hospitalisation (OR = 1.74; IC 95%: 1.41–2.14) and ICU admission (OR = 1.79; IC 95%: 1.17–2.72) and a slight increase in mortality risk, which, however, was not statistically significant (OR = 1.28; IC 95%: 0.79–2.06).

## 4. Discussion

This study, based on the large, unselected population of an entire region of Italy, showed that PWMS had the same probability of testing positive and dying from COVID-19 as the general population. They were tested more frequently for SARS-CoV-2 and had a higher probability of being hospitalised and admitted to an ICU.

Several case series have been published on the prevalence of COVID-19 infection and its outcomes in PWMS, with contradictory results. Moghadasi et al. [5] meta-analysed data from twelve articles with a number of 1394 possible/confirmed cases of PWMS and COVID-19 infection among 101,462 MS patients. The pooled prevalence of suspected COVID-19 in MS patients was 4%, the pooled prevalence of hospitalisation in infected cases was 10%, and the pooled prevalence of death in hospitalised cases was 4%. These case series recruited extremely heterogeneous patients with regard to possible risk factors for poor outcomes, including age, disease course, disability status, drugs used, and co-morbidity. Furthermore, they were collected in different periods of the pandemic, which may imply further different prevalence and probability of outcomes. These hospital-based collections of cases lacked appropriate controls, thus hampering any comparison with the general population. 

Our population-based study compares the incidence of COVID-19 infection and its outcomes in all PWMS with the general population of the same age span in the same area. A few other studies have used a similar design, although with some limitations. Eder et al. [21,22], using health administrative data from Ontario-Canada, compared adult MS patients with matched non-MS individuals from the general population and provided age- and sex-adjusted measures of risk. Despite the OR for MS patients of SARS-CoV2 testing being 1.11, the risk of SARS-CoV-2 infection was not significantly elevated (OR = 0.77). Similar to our result, the risks were higher for hospitalisation (1.77), and for a composite measure of severity (2.01), including admission to the ICU, ventilation, or death during admission (2.01). Richter et al. [10] conducted a retrospective cross-sectional study using the administrative database of all hospitalised patients diagnosed with PCR-confirmed COVID-19, comparing 551 individuals with a concurrent MS diagnosis with 156,973 without MS. When stratified by age group, the risk of ICU admission and the use of invasive or non-invasive ventilation was lower for MS compared to non-MS individuals, and in-hospital mortality was not significantly different between the two groups. Both studies, covering a large number of MS patients, did not show an increased prevalence of SARS-CoV-2 infection in PWMS. The other outcomes of our study were hardly comparable because Richter et al. limited their evaluation only to hospitalised PWMS and in-hospital mortality, and Eder et al. used an outcome combining ICU admission and in-hospital death.

Other studies identified patients in specialised MS centres or nationwide databases, where patients were recruited on a voluntary basis and may not be representative of the whole population of PWMS, thus introducing a possible selection bias. Different study designs were used, comparing the prevalence of infection and its outcomes between cohorts of PWMS and the age- and the sex-matched general population [7,23,24], or simply with COVID-19 data from the general population, without taking into account its age and sex distribution [16,17,24]. Sormani et al. [7] found an increased risk of severe events (RR = 2.12 for hospitalisation, 2.19 for ICU admission, and 2.43 for death) in an Italian cohort from MS centres. A Spanish study [23] found a lower infection incidence rate in PWMS patients than in the general population (adjusted incidence rate ratio = 0.78) but a higher hospitalisation rate (relative risk = 5.03) and also found a significantly lower case-fatality ratio in PWMS than in the general population of the same area, although this was based on only five deaths. Fernandes et al. [25] reported a similar proportion of SARS-CoV-2 positive PCR in MS patients and a slight excess of deaths, although based on only five deaths, compared to the general Scottish population. However, compared with an age- and sex-matched general population (through different linkage systems), the information obtained from these three cohorts is valid only for MS patients carrying characteristics similar to those of the cohorts and cannot be straightforwardly transferred to the whole of PWMS. 

Studies comparing outcomes in PWMS from a geographical area with non-adjusted nationwide data found a lower or similar prevalence of COVID-19 infection [16,17,24] and hospitalisation or mortality rates due to COVID-19 [14]. However, a possible bias determined by the comparison of MS cohorts recruited in a single region with nationwide data could be added to those outlined above. 

We found a significantly higher percentage of PWMS resorting to swabs. Although in Italy [18] as elsewhere [26], the use of healthcare services by PWMS during the pandemic was reduced, this may not be true for the prescription of swabs. This result is in line with what has been observed among patients with immune-mediated inflammatory diseases [21] and other chronic diseases [27,28]. Higher rates of testing may be explained by several factors, including the perception of a higher risk of COVID-19 by PWMS and their doctors, general higher health awareness of PWMS, higher attention to symptoms, and the need to test for SARS-CoV-2 before hospital admission or out-patient visits.

However, the probability to test positive was not different from the general population. Although PWMS usually experience a higher frequency of infections compared with the general population [29,30], this does not seem to apply to SARS-CoV-2 infection.

The risk of hospitalisation was overall higher for PWMS, and it was almost double for PWMS under 60 years, compared to the general population. Comorbidity with diabetes and COPD did not increase the risk of hospitalisation in PWMS. There is no clear explanation for this: it has been postulated that this may be due to the use of admission criteria that precautionary prioritised patients with chronic comorbidities and/or taking immunosuppressive medication [23]. On the other hand, as a general reduction in the hospitalisation rate occurred in Italy during the pandemic [1], we could not exclude that this affected PWMS less, thus giving the impression of an apparent increase in their hospitalisation rate. 

Once hospitalised, women with MS, but not men, had a higher risk of being admitted to the ICU. Other known risks for severe COVID-19 disease, such as age, COPD, and diabetes [31], did not substantially increase the risk of PWMS being admitted to the ICU in our cohort.

Finally, the proportion of deaths was lower among PWMS than in the general population, but after adjustment for possible confounders, we found a slightly higher risk for PWMS, although this was not statistically significant. This discrepancy is most likely due to the younger age of PWMS compared with the general population, combined with the well-known higher mortality of COVID-19 infections in the oldest [31]. Thanks to the linkage with the regional population register, we obtained information about patients who died in and outside of the hospital, either before they could be admitted or after they were discharged, which was not possible in other studies [10,22].

Age was the clearest determinant of hospitalisation, admission, and mortality in PWMS with COVID-19, mirroring what was observed in the general population [31]. A metaanalysis of 15 studies [6] showed age, male sex, disability, and comorbidity to be major determinants of poor outcomes, and studies based on clinical cohorts also added the use of CD-20 medications [6,7,16]. In our study, we did not find a clear influence of sex but confirmed that two frequent comorbidities, diabetes, and COPD, were associated with higher hospitalisation and mortality in PWMS as well, although to a lesser extent than in the general population. Unfortunately, from electronic records, we obtained neither information on the degree of disability nor on the therapies prescribed to patients, thus preventing the stratification of these two important determinants.

This is one of the few population-based studies to compare testing for SARS-CoV-2, positive testing, hospitalisation within 30 days after testing positive, admission to the ICU, and 30-day in- and out-hospital mortality between PWMS and the MS-free population. The full coverage of the entire populations of PWMS and MS-free subjects followed during the pandemic, and the complete reporting of their whole clinical paths, from swab testing to health outcomes, avoided some of the major limitations of previous studies, including selection and attrition bias, and a lack of adequate controls. 

Some limitations of our study should be considered: first, even if we used a highly reliable validated algorithm to identify MS cases from electronic health records, some misclassification could have occurred. Second, as reported above, it was impossible to stratify MS patients with respect to their drug therapy, either DMD or steroids, since this information was no more available in our administrative database; since it has been shown that COVID-19 related outcomes may differ, depending on the type of drugs taken [15,32,33], our results suffer from the inverse effects on health outcomes of the different therapies of the MS population. Additionally, we were not able to classify patients according to their level of disability, a risk factor for unfavourable outcomes, since this information was not available. However, from the perspective of public health, it is of great interest to notice that, unlike that which has been reported in other studies from Europe (including Italy) [5,7,9], PWMS resident in Piedmont suffering from MS does not have an increased risk of mortality compared to the MS-free population. Third, except for COPD and diabetes, we were not able to better describe the clinical characteristics of the two populations and to adjust for the different likelihood of severe outcomes. However, to partially overcome this issue, we adjusted for educational level, which is widely considered a proxy of comorbidities and health status [34]. Fourth, since outcomes depended largely on the policies of the tracing of infected people adopted in the local territory and on the access and quality of the care delivered, our results were only partially generalisable to other Italian regions or other countries. Lastly, it remains to be ascertained whether the rates of adverse outcomes have changed in subsequent waves of COVID-19 following the appearance of new SARS-CoV-2 variants and changes in the pattern of care in patients with COVID-19.

## 5. Conclusions

In a large population study, we have shown that PWMS experience a higher rate of testing for COVID-19 infection but have the same rate of positivity compared to the general population. PWMS with COVID-19 have an increased risk of hospitalisation and admission to the ICU, but their mortality rate does not differ from that of the general population. Therefore, the quality of care provided to patients with severe chronic conditions such as MS has remained at good levels, even during the pandemic’s highest peak.

## Figures and Tables

**Table 1:** Characteristics of the study populations.

	MS		MS-Free		
	N	%	N	%	*p* Value
**Sex**					
Female	6312	67.63	2,12,6591	51.29	<0.0001
Male	3021	32.37	2,019,265	48.71	
**Age classes**					
<30	631	6.76	933,271	22.51	<0.0001
30–39	1406	15.06	474,793	11.45	
40–49	2392	25.63	647,139	15.61	
50–59	2557	27.40	700,519	16.90	
60–69	1481	15.87	565,251	13.63	
70+	866	9.28	824,883	19.87	
**Educational level**					
Low	653	7.00	647,560	15.62	<0.0001
Middle	3836	41.10	1,402,528	33.83	
High	4593	49.21	1,268,956	30.61	
Missing	251	2.69	826,812	19.94	
**CODP**					
No	9223	98.82	4,086,427	98.57	0.0385
Yes	110	1.18	59,429	1.43	
**Diabetes**					
No	8847	94.79	3,877,900	93.54	<0.0001
Yes	486	5.21	267,956	6.46	

**Table 2:** Crude prevalence of the variables by each of the five outcomes.

	Swab			Positivity			Hospitalization			Intensive Care Unit			Mortality		
	MS	MS-Free	*p*-Value	MS	MS-Free	*p*-Value	MS	MS-Free	*p*-Value	MS	MS-Free	*p*-Value	MS	MS-Free	*p*-Value
**Number**	3540	1,340,433		868	325,803		116	38,179		23	6471		19	12,004	
	%	%		%	%		%	%		%	%		%	%	
**Total**	37.93	32.33	<0.0001	24.52	24.31	0.7669	13.36	11.72	0.1323	2.65	1.99	0.1619	2.19	3.68	0.0194
**Sex**															
Female	39.16	33.46	<0.0001	24.19	24.26	<0.0001	11.20	9.06	0.0003	2.68	1.13	<0.0001	1.67	3.09	0.4525
Male	35.35	31.14		25.28	24.35		18.15	14.63		2.59	2.94		3.33	4.40	
**Age classes**															
<30	44.06	34.16	<0.0001	23.74	21.01	<0.0001	1.52	0.79	<0.0001	0.00	0.06	0.1301	0.00	0.01	0.0002
30–39	43.39	35.88		21.80	22.57		3.01	1.99		0.00	0.25		0.00	0.03	
40–49	39.88	33.82		23.17	25.53		5.43	4.11		0.90	0.71		0.00	0.16	
50–59	36.72	33.26		25.24	26.15		17.30	8.86		3.80	1.88		1.27	0.54	
60–69	30.25	27.41		25.22	25.06		23.89	19.26		5.31	4.84		5.31	2.97	
>=70	35.91	28.94		31.51	26.49		31.63	33.32		6.12	4.48		10.20	16.19	
**Educational level**															
Low	33.23	30.66	<0.0001	29.95	27.41	<0.0001	27.69	25.23	<0.0001	3.08	3.36	0.1237	6.15	11.84	0.0130
Middle	35.32	31.51		27.31	26.07		15.95	12.10		3.24	2.28		2.97	2.97	
High	41.06	37.33		22.00	23.45		8.92	8.00		2.17	1.61		0.96	1.54	
Missing	32.67	27.35		21.95	19.93		11.11	3.59		0.00	0.50		0.00	0.93	
**COPD**															
No	37.91	32.23	0.0213	24.54	24.28	0.1322	13.05	11.16	0.2220	2.68	1.91	0.2537	2.10	3.43	0.5586
Yes	40.00	39.50		22.73	25.53		40.00	39.32		0.00	5.76		10.00	18.34	
**Diabetes**															
No	37.95	32.18	<0.0001	24.19	24.07	0.1382	12.56	9.93	0.0126	2.71	1.66	0.0346	2.09	2.90	0.0919
Yes	37.65	34.52		30.60	27.48		25.00	32.40		1.79	5.82		3.57	13.22	

## Data Availability

The data presented in this study are available on request from the corresponding author. The data are not publicly available due to strict privacy issue.
